# Clinicopathological characteristics and risk factors for recurrence of well-differentiated pancreatic neuroendocrine tumors after radical surgery: a case-control study

**DOI:** 10.1186/s12957-019-1606-8

**Published:** 2019-04-11

**Authors:** Pan Zhang, Yuan-liang Li, Xu-dong Qiu, Jie Luo, Yan-fen Shi, Yong-liang Sun, Fei Su, Zhi-rong Qi, Huang-ying Tan

**Affiliations:** 1 Beijing University of Chinese Medicine, Beijing, China; 2 Department of Pathology, China-Japan Friendship Hospital, Beijing, China; 3 Department of General Surgery, China-Japan Friendship Hospital, Beijing, China; 4 Department of Integrative Oncology, China-Japan Friendship Hospital, 2 Yinghuadong Road, Chaoyang District, Beijing, 100029 China

**Keywords:** Recurrence, Well-differentiated, PanNETs, Postoperative, Risk factor

## Abstract

**Background:**

Well-differentiated pancreatic neuroendocrine tumors (PanNETs) usually have a good prognosis; however, there are patients that experience recurrence after curative resection.

**Aim:**

To explore recurrence-related risk factors by analyzing clinicopathological data of PanNETs after radical surgery.

**Methods:**

Clinical and pathological data from 47 patients with well-differentiated PanNETs at China-Japan Friendship Hospital from January 2012 to March 2016 were analyzed retrospectively. Univariate and multivariate analyses of the risk factors of PanNETs for postoperative recurrence were conducted.

**Results:**

Among the 47 patients with well-differentiated PanNETs, there were 38 cases with non-functioning tumors, 9 cases with functional tumors (6 insulinomas, 1 gastrinoma, 1 glucagonoma, and 1 VIPomas). There are 17 cases (36.2%) in the pancreatic head, 17 (36.2%) in the body and tail, 9 (19.1%) in the tail, and 4 (8.5%) in the body. The median tumor size was 3.65 (IQR 2–5.5) cm. Fourteen cases (29.8%) were NET G1, and 33 cases (70.2%) were NET G2. In regard to the clinical stage, 9 (19.1%) cases were IA, 14 (29.8%) cases were IB, 7 (14.9%) cases were IIA, 14 (29.8%) cases were IIB, and 3 cases unknown. There were 17 patients who presented with postoperative recurrence. Univariate analysis showed that AJCC TNM staging, Ki67 index, vascular invasion, margin status, and the regional stage of the tumors are related to the recurrence of patients with PanNETs (*p* < 0.05). The results of multivariate analysis showed that Ki67 index ≥ 10% is an independent risk factor for the postoperative recurrence of PanNETs (*p* < 0.05).

**Conclusion:**

The Ki67 index ≥ 10% is an independent risk factor for recurrence in well-differentiated PanNETs after radical surgery, and close surveillance for these patients may be needed.

## Background

Neuroendocrine neoplasm (NEN) is a rare and heterogeneous tumor type that originates in peptidergic neurons and neuroendocrine cells. The pancreas is a common site for neuroendocrine tumors. The Surveillance, Epidemiology, and End Results (SEER) database shows that gastroenteropancreatic neuroendocrine neoplasms (GEP-NENs) are most commonly observed in the rectum, followed by the jejuno-ileum, pancreas, and stomach [1]. Well-differentiated pancreatic neuroendocrine tumor (PanNET) usually has a good prognosis. However, postoperative recurrence or metastasis of well-differentiated PanNET is not rare in the clinic, most of the studies have been retrospective, and the risk factors of recurrence differ [2–5]. The aim of this study was to investigate the clinicopathological data of patients with well-differentiated PanNETs after radical surgery and to analyze the risk factors of the postoperative recurrence of well-differentiated PanNETs to provide clinical guidance.

## Methods

The study examined 47 well-differentiated PanNETs after surgery in China-Japan Friendship Hospital in Beijing, from January 2012 to December 2016, with the following inclusion criteria: (1) patients who were diagnosed with NET G1 or NET G2 pancreatic neuroendocrine tumors by surgical pathology according to the 2010 version of the WHO grading after curative resection [6]; (2) patients who were confirmed to have no distant metastasis at the time of diagnosis and who were in a disease-free state after radical operation; and (3) patients who only had one primary tumor (but multiple lesions could be found in one origin) and received surgical resection at the primary site were included in this study.

PanNETs related to multiple endocrine neoplasia type 1 (MEN-1) or with other malignant tumors were excluded from this study. All patients provided full informed consent.

### Clinical materials

All patients underwent a routine evaluation that included clinical, laboratory, and radiological imaging. Radiological imaging included ultrasonography (US), abdominal computed tomography (CT), and/or magnetic resonance imaging (MRI). Endoscopic ultrasonography and/or octreotide scintigraphy (Octreoscan/ 68 Ga PET-CT) was performed in some patients as part of pre-operative work up. All the patients were free of distant metastatic disease at diagnosis and not associated with a genetic predisposition for the development of PanNETs (like MEN-1). Depending on the tumor location, different surgery procedures were chosen. Pathology reports were reviewed for the diagnosis of PanNET and the Ki67 index.

General information and clinical, surgical, pathological, biochemical, and radiological data were retrospectively collected. Further, the size, location, margin status, pathological grade, the status of vascular and perineural invasion, and Ki67 index (%) of tumors were described in detail in this study, together with the features of the localized or regional stage PanNENs. If these data were not described by surgical pathology, the preoperative radiological data were referenced.

TNM staging classification was based on the seventh edition of TNM staging of pancreatic neuroendocrine tumors released by the seventh American Cancer Society, i.e., the American Joint Committee on Cancer (AJCC), including primary tumor (T), regional lymph nodes (N), and distant metastases (M) staging [7]. PanNETs were divided into three categories using the 2010 WHO digestive system tumor classification criteria [8].

Recurrence was defined as local recurrence in the pancreas, new localization in lymph nodes, or the development of distant metastases. Localized-stage PanNETs were defined as invasive neoplasms confined entirely to the organ of origin. Regional-stage PanNETs were defined as neoplasms that extended beyond the limits of the organ of origin, directly into surrounding organs or tissue and/or involving regional lymph nodes. Vascular invasion was defined as microscopic invasions (under the microscope, cells that are actually getting into blood vessels, this gives us some indication that these cells may have a tendency to spread).

### Follow-up

The deadline for follow-up was December 2018. Patients in the study were assessed by radiological examination (computed tomography (CT) and magnetic resonance imaging (MRI)) every 6 months after surgery to determine whether there was recurrence or metastasis of the tumors. Some patients underwent somatostatin receptor scintigraphy (Octreoscan) or Ga68 PET-CT as part of their follow-up assessment. The results of the assessment were recorded, including the survival status of all patients, recurrence time of recurred patients, and the last follow-up time (the last imaging time) for patients without recurrence respectively.

### Statistical methods

Data were expressed as the median and 25th–75th interquartile ranges (IQRs). Disease-free survival (DFS) was defined as the length of time after primary treatment for cancer while the patient survived without any signs or symptoms of cancer. The Kaplan-Meier method and log-rank test were used to evaluate suspicious risk factors and to compare recurrence rates. The univariate analysis of the risk factors used the log-rank test and the Cox regression test. The multivariate analysis of the risk factors used Cox proportional-hazard regression analysis and forward selection (likelihood ratio). Hazard ratios (HRs) and the corresponding 95% confidence intervals (CIs) were recorded. All *p* values were bidirectional. *p* < 0.05 was statistically significant. SPSS 20.0 was used for statistical analysis.

## Results

### Clinical features

There were 47 patients in this study, 17 males and 30 females, and the median age was 50 years old (IQR 44–60 years). The median follow-up time was 49 months (IQR 31–62 months). Thirty-eight cases (80.9%) were non-functional PanNETs, and 9 cases (19.1%) were functional PanNETs (6 insulinomas, 1 vasoactive intestinal peptide (VIP) tumor, 1 glucagonoma, and 1 gastrinoma). Twenty-one patients had no symptoms, 6 patients had hypoglycemia with paroxysmal loss of consciousness as the main symptom, 4 patients had abdominal pain and abdominal distension, 2 patients had diarrhea, 1 patient had sour regurgitation as the main symptom, 1 patient had facial flushing, 1 patient had weight loss, and 1 patient had angular cheilitis and erythema on the face and both legs. Among the 47 cases, 17 cases (36.2%) occurred in the pancreatic head, 17 (36.2%) occurred in the body and tail, 9 (19.1%) occurred in the tail, and 4 (8.5%) occurred in the body.

The tumor sizes ranged from 0.3 cm to 13 cm. The median tumor size was 3.65 (IQR 2–5.5) cm. A total of 34 cases (72.3%) had tumors ≥ 2 cm, 10 cases (21.3%) had tumors < 2 cm, and the size of the tumors for 3 patients was unknown. In this study, two patients had multiple pancreatic neuroendocrine tumor lesions (the number of lesions was 2), while the remaining patients had a single lesion. The mean size of lesions in a symptomatic patient is 3.55 cm and 4.56 cm in asymptomatic patient. The tumor size between these two groups shows no significant difference (*p* = 0.173).

### Pathological features

Among the 47 cases, 14 cases (29.8%) were G1, and 33 cases were G2 (70.2%). The median Ki67 index was 5% (IQR 2–7%), 39 cases (83.0%) were less than 10% in Ki67, and 8 cases (17.0%) were greater than or equal to 10%. A total of 21 cases (44.7%) had Ki67 index of less than 5%, and a total of 26 cases (55.3%) had Ki67 index of greater than or equal to 5%. The clinical staging in these 47 cases were as follows: T1, 9 cases (19.1%); T2, 20 cases (42.6%); T3, 15 (31.9%); and 3 cases were unknown; N0, 34 cases (72.3%); N1, 12 cases (25.5%); and 1 case was unknown; stage IA, 9 cases (19.1%); stage IB, 14 cases (29.8%); stage IIA, 7 cases (14.9%); stage IIB, 14 cases (29.8%); and 3 cases unknown. A total of 23 cases (48.9%) were localized-stage PanNETs, 23 cases (48.9%) were regional-stage PanNETs, and 1 case was unknown. Among the regional-stage PanNETs, 11 patients (23.4%) had tumors that extended beyond the pancreas, 33 cases (70.2%) had tumors within the pancreas, and the remaining 3 cases were not clear; 12 cases (25.5%) invaded peripheral tissues, 24 cases (72.3%) did not invade peripheral tissues, and 1 case was unknown.

In regard to other pathological features, there were 7 cases (14.9%) with vascular invasion, 38 cases (80.9%) without vascular invasion, and the status of vascular invasion for 2 cases was unknown. There were 4 cases (8.5%) with perineural invasion, 42 cases (89.4%) without perineural invasion, and 1 case was unknown. Eight cases (17.0%) had positive margins, 38 cases (80.9%) had negative margins, and 1 case (4.3%) had unknown state of margins. The patients’ clinical and pathological features were summarized in Table 1.

**Table 1:** Clinicopathological features of 47 postoperative patients with recurrent and non-recurrent PanNETs

		Non-recurrent PanNETs	Recurrent PanNETs	All**
Age ,median, years		51.5	49	50
Gender, *n* (%)	Male	9(22.0)	5(12.2)	17(36.2)
	Female	15(36.6)	12(29.3)	30(63.8)
Tumor function, *n* (%)	Functional	5(12.2)	2(4.9)	9(19.1)
	Non-functional	19 (46.3)	15(36.6)	38(80.9)
Tumor location, *n* (%)	Body and tail	6(14.6)	9(22.0)	17(36.2)
	Head	9(22.0)	5(12.2)	17(36.2)
	Body	3(7.3)	0(0.0)	4(8.5)
	Tail	6(14.6)	3(7.3)	9(19.1)
Tumor size, median (cm)		5	8.2	3.65
T	T1	7(18.4)	0(0.0)	9(19.1)
	T2	10(26.3)	6(15.8)	20(42.6)
	T3	7(18.4)	8(21.1)	15(31.9)
*N* (lymph nodes status), *n* (%)	Negative (N0)	19(47.5)	10(25.0)	34(72.3)
	Positive (N1)	5(12.5)	6(15.0)	12(25.5)
Stage, *n* (%)	IA	7(18.4)	0(0.0)	9(19.1)
	IB	9(23.7)	2(5.3)	14(29.8)
	IIA	4(10.5)	3(7.9)	7(14.9)
	IIB	4(10.5)	9(23.7)	14(29.8)
Grade, *n* (%)	G1	6(14.6)	2(4.9)	14(29.8)
	G2	18(43.9)	15(36.6)	33(70.2)
Ki67 index, median,%		5	8.2	5
Ki67 index	≥ 10%	5(12.2)	3(7.3)	39(83.0)
	< 10%	21(51.2)	12(29.3)	8(17.0)
Vascular invasion, *n* (%)	Positive	1(2.6)	6(15.4)	7(14.9)
	Negative	23(59.0)	9(23.1)	38(80.9)
Perineural invasion, *n* (%)	Positive	0(0.0)	4(10.0)	4(8.5)
	Negative	24(60.0)	12(30.0)	42(89.4)
Margin status, *n* (%)	Positive	2(5.0)	6(15.0)	8(17.0)
	Negative	22(55.0)	10(25.0)	38(80.9)
Localized or regional stage tumors, *n* (%)	Regional	8(20.0)	14(35.0)	23(48.9)
	Localized	16(40.0)	2(5.0)	23(48.9)

*6 patients lost to follow-up and some clinical data is not clear are not be included in this table.

**The size of the tumor and clinical staging for 3 patients were unknown; vascular tumor thrombus in 2 cases were unknown, perineural invasion and margin status in 1 case is unknown

### Radical surgery and postoperative treatments

Fourteen patients underwent a pancreaticoduodenectomy (29.8%), 23 patients (48.9%) underwent pancreatic body and tail (with splenectomy) resection, and 10 (21.3%) cases underwent local resection. In this study, 4 cases received somatostatin analogs (SSAs), and 2 cases received gemcitabine as adjuvant therapy in local hospitals. Three cases (75%) in the SSA subgroup recurred during the follow-up period. The postoperative treatments were summarized in Table 2.

**Table 2:** The patients accepted postoperative adjuvant therapy in this study

No.	Whether relapse	DFS (mouths)	Treatment	Ki67 index (%)
1	No recurred	18	Octreotide	5
2	Recurred	22	Octreotide	5
3	Recurred	17	Octreotide	15
4	Recurred	9	Chemotherapy (gemcitabine)	5
5	Recurred	13	Octreotide	4
6	Recurred	72	Chemotherapy (gemcitabine)	3

### Recurrence features

In our study, a total of 17 cases recurred during follow-up. In particular, 2 cases were insulinoma. In recurred patients, 11 cases had liver metastases, 5 of which had lymph node metastasis (including 2 of the cases of liver metastases), and 1 of which had metastasis in the pancreatic stump. In the recurrent cases, 11 cases were diagnosed via abdominal CT, 4 cases were found by MRI, and 2 cases were found by somatostatin receptor scintigraphy (SRS). In addition, 2 patients died, and 6 patients (10.6%) lost contact during the follow-up period. In this study, the recurrence rate was 36.2%, and the median DFS was 29 months (IQR 21–47.5 months). The clinical features of 17 recurred patients were summarized in Table 3.

**Table 3:** Clinical feature of 17 recurred patients

Number	Gender	Age (years)	Tumor functional type	Ki67 index	Tumor size (cm)	Positive lymph nodes	Staging	Localized or regional tumors	Vascular invasion	Perineural invasion	DFS (mouth)
1	M	51	NF	5	4.5	−	IIA	Regional	Unknown	−	20
2	F	46	NF	15	8	−	IIB	Regional	+	+	18
3	F	52	NF	5	4.5	−	IIB	Regional	−	−	23
4	M	48	NF	10	3	+	IIB	Regional	−	−	7
5	F	68	NF	7	Unknown	−	Unknown	Regional	Unknown	−	6
6	F	65	Insulinoma	20	5	+	IIB	Regional	−	−	6
7	F	72	NF	20	3	−	IIB	Regional	−	−	6
8	F	65	NF	5	4	−	IIB	Localized	−	+	22
9	F	47	NF	3	3	+	IIB	Regional	−	−	26
10	M	47	NF	5	3.3	+	IIB	Regional	+	−	23
11	F	43	NF	3	5.5	−	IIA	Regional	−	−	72
12	F	29	Insulinoma	2	Unknown	Unknown	Unknown	Unknown	Unknown	Unknown	118
13	M	63	NF	15	5	−	IIA	Regional	+	+	17
14	F	54	NF	5	13	−	IIA	Regional	+	−	9
15	F	61	NF	5	4.5	−	IB	Localized	−	−	72
16	M	23	NF	7	5	+	Unknown	Regional	+	−	15
17	F	23	NF	1	7	+	IIB	Regional	−	+	33

### Univariate analysis and multivariate analysis of the recurrence of postoperative PanNETs

Univariate analysis was conducted with multiple factors, such as age, sex, tumor function, surgical procedures, tumor location, tumor size, AJCC TNM stage, WHO grade, Ki67 index, localized or regional stage, perineural invasion, vascular invasion, and margin status, using log-rank analysis and Cox regression analysis. Univariate analysis conducted with the log-rank test showed that surgical procedures, tumor size (≥ 2 cm), TNM stage, Ki67 index (greater than 10% and greater than 5%), vascular invasion, margin status, and regional-stage tumors were factors that were related to the postoperative recurrence of PanNETs. Cox regression univariate analysis showed that TNM stage, Ki67 index, vascular invasion, margin status, and regional-stage tumors were factors related to recurrence (see Table 4).

**Table 4:** Risk factors for DFS at the univariate analysis (*n* = 47 patients)

		Log-rank		COX regression	
		3-year non-recurrent rate %	*P*	*P*	Exp(B) (95% CI)
Age	≧ 50 years	46.6	0.346	0.353	0.604(0.208–1.753)
	< 50 years	50.1			
Gender	Male	49.2	0.862	0.863	0.908(0.301–2.743)
	Female	47.5			
Tumor function	Functional	80.0	0.197	0.226	0.280(0.036–2.198)
	Non-functional	41.6			
Surgical type	Pancreatico -duodenectomy	26.3	0.031	0.055	0.481(0.227–1.016)
	Pancreatic body and tail resection	32.0			
	Local resection	100			
Tumor location	Body and tail	28.3	0.693	0.422	0.824(0.514–1.322)
	Head	39.7			
	Body	100			
	Tail	77.8			
Tumor size	≧ 2 cm	33.5	0.078	0.274	28.199(0.071–11218.74)
	< 2 cm	100			
Tumor size	≧ 4 cm	38.9	0.376	0.384	1.689(0.518–5.503)
	< 4 cm	58.5			
Stage	IA	100	0.008	0.007	3.081(1.366–6.947)
	IB	87.5			
	IIA	64.3			
	IIB	0			
Grade	G1	75	0.029	0.061	7.011(0.917–53.608)
	G2	38.6			
Ki-67	≧ 10%	19.4	0.000	0.003	7.585(2.018–28.5)
	< 10%	54.9			
Ki-67	≧ 5%	38.1	0.038	0.053	3.538(0.986–12.695)
	< 5%	60.6			
Lymph nodes status	Positive	17.0	0.210	0.221	0.516(0.179–1.490)
	Negative	58.5			
Vascular invasion	Positive	15.2	0.024	0.032	0.305(0.103–0.905)
	Negative	54.1			
Perineural invasion	Positive	0	0.073	0.087	0.36(0.112–1.159)
	Negative	56.6			
Margin status, *n* (%)	Positive	15.6	0.029	0.039	3.057(1.057–8.840)
	Negative	56.2			
Localized or regional stage tumors	Regional	20.0	0.007	0.018	0.165(0.037–0.734)
	Localized	85.7			

**Fig. 1:**
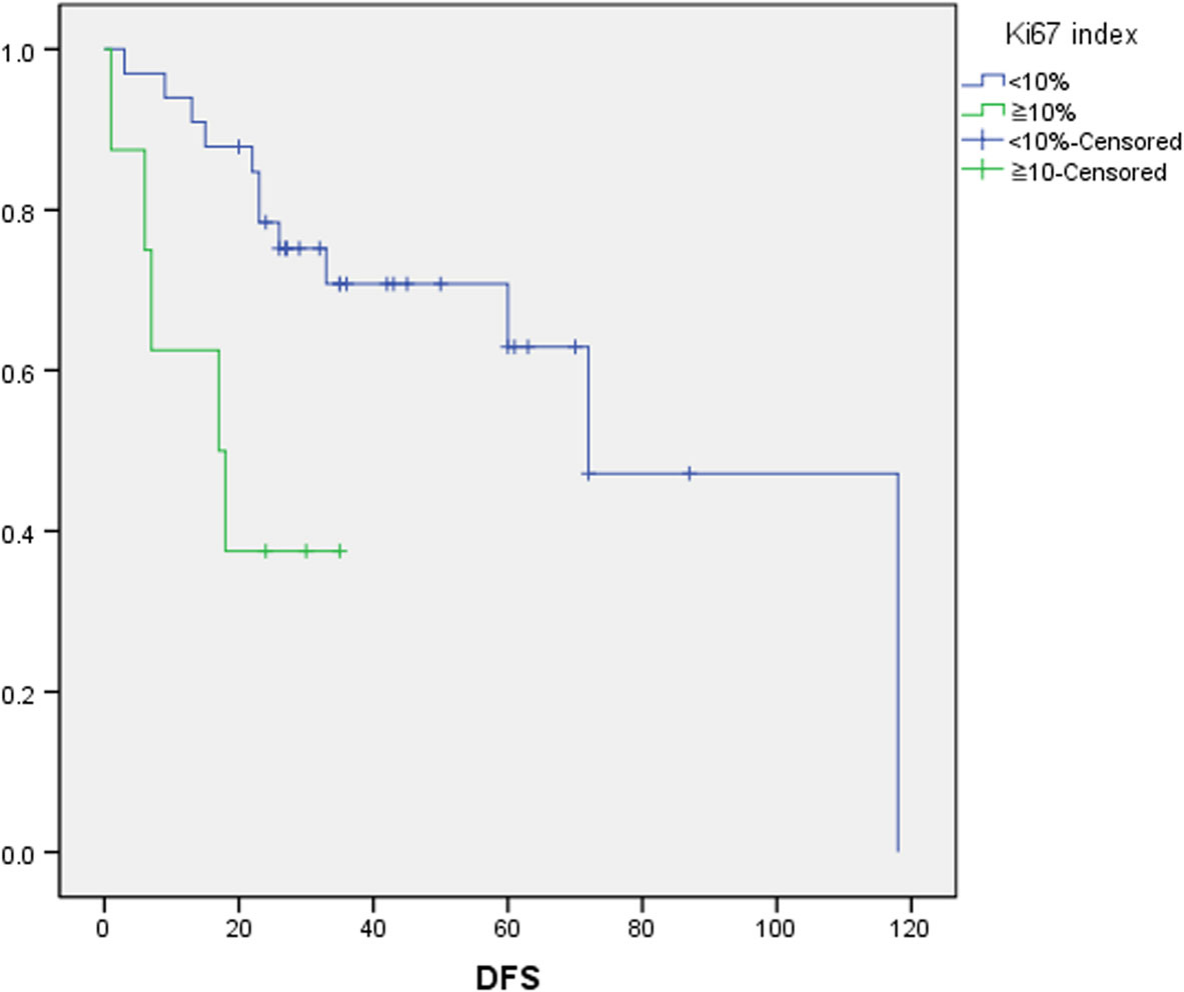
Kaplan-Meier curve for DFS of postoperative PanNETs by Ki67 index. Thirty-nine cases were less than 10%, 8 cases were greater than or equal to 10%, and DFS of these 2 groups have a statistical difference (*p* < 0.05)

Multivariate analysis applying Cox proportional-hazard regression analysis was used to analyze the clinical pathological factors for which *p* < 0.05 in both the log-rank test results and Cox regression. The results of multivariate analysis showed that Ki67 index of greater than 10% was an independent risk factor for the postoperative recurrence of PanNETs (*p* < 0.05), as shown in Table 5. Kaplan-Meier survival analysis of disease-free survival (DFS) showed that the Ki67 index had statistical significance for the recurrence of postoperative PanNETs (*p* < 0.05), and the result was shown in Fig. 1.

**Table 5:** Multivariate analysis of the recurrence-related factors of postoperative PanNETs

	*B*	SE	Wald	df	Sig.	Exp(*B*)	95.0% CI for Exp(*B*)	
							Lower	Upper
Ki67 index	2.670	0.842	10.057	1	0.002	14.440	2.773	75.201

## Discussion

Recent guidelines [9, 10] recommend surveillance with evaluation every 6–12 months for well-differentiated PanNET patients after surgery for up to 10 years but did not recommend postoperative adjuvant therapy. Identifying high-risk factors for recurrence through a retrospective study is the first step to achieving good outcomes. Previous studies had made some contributions. Boninsegna, L et al. showed that the lymph node ratio and Ki67 index are predictors of recurrence after resection for malignant PanNETs [3]. Genc C. G and colleagues suggested that patients at high risk for recurrence after curative resection of G1 or G2 PanNETs can be identified by the Ki67 index which is higher than 5% [11]; additionally, Genc C. G and colleagues presented a new scoring system that could predict recurrence after curative resection of grades 1 and 2 NF-PNETs [2]. Gao H et al. presented a novel risk stratification of recurrence for resected pancreatic neuroendocrine tumors to identify PanNET patients with different risk of recurrence [4]. Yao et al. [12] suggested that surveillance past 5 years may be avoided in elderly patients with a low risk of recurrence, and pancreatic, higher grade, and regional stage NETs were high-risk factors for recurrence; however, no relevant adjuvant treatment was mentioned in their study. In addition, the 2010 WHO classification [6] and the degree of the cystic component of PanNETs [13] were reported to be recurrence-related risk factors for postoperative PanNETs.

In our study, 17 cases presented with recurrence or metastasis after radical surgery, and the recurrence rate was 36.2%, compared with the 8.5–36.3% recurrence rates in other studies [2, 4, 11, 14, 15]. The higher recurrence rate at our center may be because we had a smaller sample size; we performed a single-center study, and the follow-up period was somewhat short. Based on clinical experience and relevant articles, we chose 14 factors that we used to perform univariate analysis, and the results showed that AJCC TNM stage, Ki67 index (greater than 10%), vascular invasion, margin status, and regional-stage tumors had statistical significance in the univariate analysis. Multivariate analysis showed that Ki67 index of greater than 10% was an independent risk factor for postoperative recurrence of PanNETs (*p* < 0.05).

The Ki67 index was usually related to the degree of the malignancy of the tumor. Consensus guidelines used Ki67 index of 2% and 20% as cut-off values to separate G1, G2, and G3. However, 2 to 20% was a large range, and the cut-off of 10% was often used by oncologists, demonstrating the heterogeneity in the malignant potential within one WHO grading group. Previous literature chose 5% and 10% as the cut-off values [3, 11, 16]. In this study, two Ki67 indexes, 5% and 10%, were selected as cut-off values, and the results showed that Ki67 index of ≥ 10% is an independent risk factor for postoperative recurrence of PanNETs, while Ki67 index of 5% was the cut-off value for statistical significance in the univariate analysis but not in the multivariate analysis. This outcome was different from the previous literature [3, 11], which showed that patients at high risk for recurrence after curative resection of G1 or G2 PanNETs can be identified by Ki67 rate of higher than 5%. According to the results of this study, patients with Ki67 index of higher than 10% may be at high risk of recurrence and should at least be more closely monitored in the follow-up period.

At present, small NF-PanNETs (< 2 cm) [2, 17] and most insulinomas (> 90%) [18] may be treated as benign tumors in consensus guidelines. However, the definition of a benign tumor is ambiguous. For example, in 2012 European Neuroendocrine Tumor Society (ENETS) guidelines [17], NF-PanNET which tumor size < 2 without metastases, invasion, angioinvasion, and Ki67 index usually around 2% belongs to a benign tumor. It seems that tumor size (≥ 2 cm or < 2 cm) is a primary criterion to distinguish the biological behavior of NF-PanNET. Meanwhile, in similar research [2], patients with NF-pNET < 2 cm were considered to have indolent recurrence pattern and were performed separate analyses. However, PanNET which tumor size is ≥ 2 cm still contains both benign and low-grade malignant; it is confusing to distinguish the benign and low-grade malignant.

In our opinion, all neuroendocrine tumors have malignant potential. Because of early detection, the NET G1 and G2 tumor which removed at an early stage (usually very small), may show a more indolent recurrence pattern, partial patients in this condition may treat as cured, i.e., benign tumors. However, some research showed that even in this so-called benign tumors, recurrences and metastases were not rare [19, 20]. In this research, there are 10 cases with tumor <2 cm (3 insulinomas included), but no 1 in this subgroup showed recurred or metastasized during follow-up. It seems that PanNET <2 cm may have a more indolent recurrence pattern; however, in univariate and multivariate analysis, the tumor size (<2 cm) shows no statistical significance in this research. Besides, T-stage standard of the ENETS staging system used a 4-cm tumor size as the cut-off value, and univariate analysis results using this cut-off still showed that tumor size was not a contributing factor to postoperative recurrence. Furthermore, two patients developed disease recurrence after resection of insulinoma in this study, at 6 and 118 months after initial surgery. In particular, one patient recurred at 6 months after surgery with a tumor size of 5 cm, but the Ki67 index was 2%. It suggests that risk factors should be comprehensively considered and we cannot generally define small NF-PanNET or insulinoma as benign tumor.

In regard to factors such as perineural invasion, vascular invasion, positive margin, and surgical procedures, a number of studies had shown that these factors are associated with survival, prognosis, and postoperative recurrence [21–24]. The univariate analysis of this study showed that vascular invasion and margin positivity were relevant risk factors for the postoperative recurrence of PanNETs (*p* < 0.05), while perineural invasion and surgical procedures had no statistical significance (*p* > 0.05). Multivariate analysis showed that perineural invasion, vascular invasion, and positive margins did not have a significant impact on the postoperative recurrence of PanNETs. This outcome may be due to the small number of objectives, and the follow-up period is not very long in this study. In addition, different derivative procedures and duct occlusion in the management of the pancreatic stump had different surgical and survival outcomes [25, 26].

This study has limitations that should be mentioned. This study was a retrospective cohort study, the number of cases included in this study was small, and the follow-up period was somewhat short. Firstly, Ki67 index was the most important risk factor for the recurrence of postoperative PanNENs, but the postoperative adjuvant therapy of PanNENs was not deeply discussed. Secondly, the serum chromogranin A data was not showed in this study, as an essential laboratory test until now. What role adjuvant therapy should play in the management of well-differentiated resected NETs is still an unanswered question, and the design and initiation of adjuvant treatment are currently unclear. Hence, multicenter and prospective studies are required in the future.

## Conclusion

Patients with well-differentiated PanNETs have longer survival times and better prognoses. It is highly recommended that all lesions should receive radical resection if the tumors are in an early stage. Based on univariate and multivariate analysis of the risk factors that impact postoperative recurrence in this study, Ki67 index ≥ 10% is an independent risk factor for the postoperative recurrence of PanNETs. Therefore, this study suggests that well-differentiated PanNET patients with high-risk factors for recurrence, especially when the Ki67 index ≥ 10%, should be closely followed up, and the role of postoperative adjuvant therapies remains unclear. Multicenter and prospective studies should be conducted to identify new and more reliable biomarkers of recurrence and appropriate postoperative adjuvant therapy.

## Abbreviations

PanNETs: Pancreatic neuroendocrine tumors
NENs: Neuroendocrine neoplasms
GEP-NENs: Gastroenteropancreatic neuroendocrine neoplasms
IQR: Interquartile range
CT: Computed tomography
MRI: Magnetic resonance imaging
NF-PNETs: Non-functional pancreatic neuroendocrine tumors
ENETS: European Neuroendocrine Tumor Society
AJCC: American Joint Committee on Cancer
